# Surface Temperature Mapping of the University of Northern Iowa Campus Using High Resolution Thermal Infrared Aerial Imageries

**DOI:** 10.3390/s8085055

**Published:** 2008-08-25

**Authors:** Alexander Savelyev, Ramanathan Sugumaran

**Affiliations:** Department of Geography, University of Northern Iowa, Cedar Falls, Iowa, USA; E-mail: savelyev@uni.edu (Alexander Savelyev)

**Keywords:** Thermal, infrared, aerial, remote sensing, buildings, steam lines, surface temperature

## Abstract

The goal of this project was to map the surface temperature of the University of Northern Iowa campus using high-resolution thermal infrared aerial imageries. A thermal camera with a spectral bandwidth of 3.0-5.0 μm was flown at the average altitude of 600 m, achieving ground resolution of 29 cm. Ground control data was used to construct the pixel- to-temperature conversion model, which was later used to produce temperature maps of the entire campus and also for validation of the model. The temperature map then was used to assess the building rooftop conditions and steam line faults in the study area. Assessment of the temperature map revealed a number of building structures that may be subject to insulation improvement due to their high surface temperatures leaks. Several hot spots were also identified on the campus for steam pipelines faults. High-resolution thermal infrared imagery proved highly effective tool for precise heat anomaly detection on the campus, and it can be used by university facility services for effective future maintenance of buildings and grounds.

## 1. Introduction

Heat loss detection is an important aspect of infrastructure maintenance. With fuel prices rising and ecology concerns gaining voice, managers and engineers face the problem of heat loss reduction. One of the common ways to improve energy efficiency is to identify temperature anomalies (“hot spots”) in the existing infrastructure. Defects in building insulation, leakage in underground pipelines and general insulation deterioration are good examples of heat loss sources. Thermal radiation is not perceived by the human eye, which complicates the process of hot spot detection. Thermal remote sensing makes thermal radiation visible, which makes it an excellent solution to the problem of locating hot spots. Thermal infrared sensors can be handheld or fixed on a platform such as an airplane, a satellite or a car. Each of these platforms has its own advantages and disadvantages.

Ground level surveys are very flexible and can easily be performed on demand. They are, however, prohibitively time-consuming when dealing with a large study region [1]. Another problem is that the results of the ground survey cannot be easily integrated into modern GIS systems. Airborne and satellite-based surveys, on the other hand, provide data that can be easily georeferenced, imported into any GIS system and prepared for automated analysis as described by MacKay [2].

Satellite systems have a number of advantages: they offer vast coverage, collect imagery at regular intervals and at lower cost. On the other hand, satellite imagery is not available on request because the orbital path is not easily adjustable. Also, the combination of limited pixel dwell-time and low level of signal in the infrared part of the spectrum only allows for spatial resolution of 60m or worse (60m for Landsat, Band #6 and 90m for Aster, Bands #10 to 14, according to NASA specifications). Finally, sun-synchronous satellites (such as Aster and Landsat) are not suitable for precise heat loss analysis, because it is the heat coming from inside the buildings that is of interest, not the re-emitted heat coming from the sun.

Aerial imagery can be acquired on request (at any time of day and night) and with high spatial resolution because of the much lower altitude of flight. Several papers have demonstrated that aerial imagery can produce temperature measurements accurate to within 0.6-1.7°C [3-5]. A number of drawbacks, such as long deployment time (the plane needs to travel to the place of survey) and relatively high cost (costs of $200,000 for bigger projects are not unusual [6]) may become a barrier for smaller projects. However, the advantages of aerial imagery make it ideal for on-demand high- resolution thermal infrared surveys.

The goal of this project was to obtain aerial thermal infrared imagery for the campus of the University of the Northern Iowa and assess its usefulness from the position of temperature anomalies detection. High spatial resolution of the imagery, fully digital image-processing framework and an attempt to perform GIS-based analysis would differentiate this survey from other papers on aerial- based infrared thermography. Analog, low-resolution infrared sensors and the lack of GIS integration significantly limit the potential of the thermal infrared research.

## 2. Data Collection

### 2.1 Study Area

**Figure 1.**
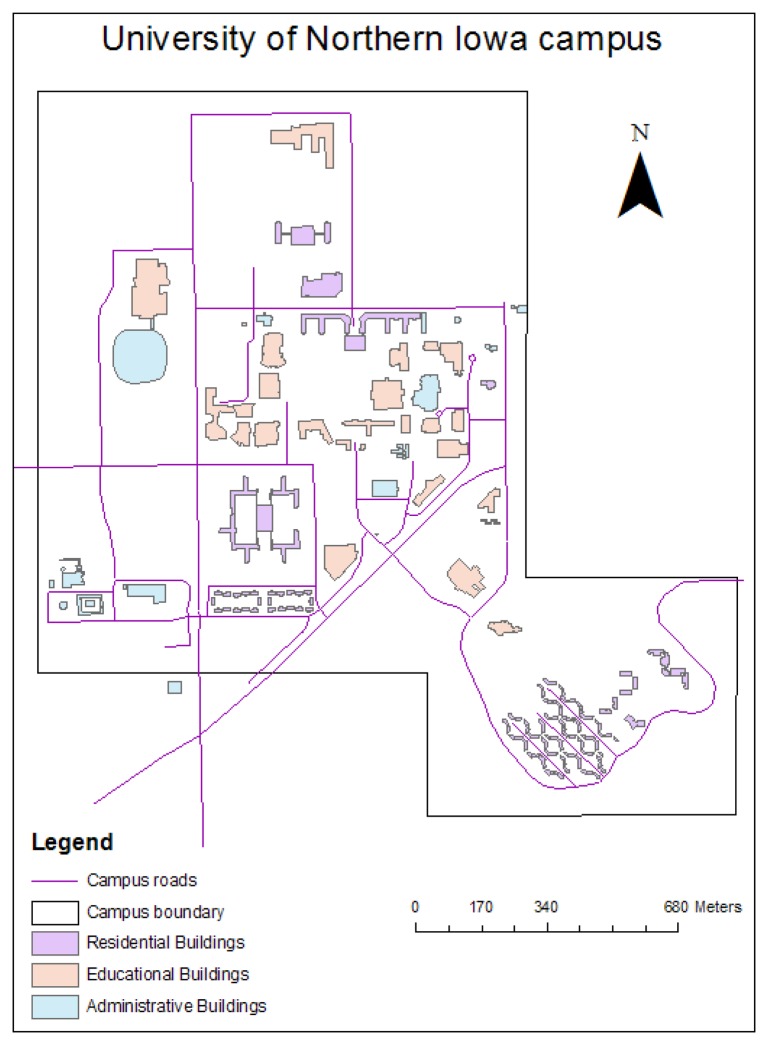
UNI campus map.

The study area for this survey was the Campus of the University of Northern Iowa (UNI) in Cedar Falls, Iowa, USA (Figure 1). Despite its small area (about 2 km sq.), the UNI campus represents a wide variety of objects of interest, including flat rooftops, sloped rooftops, underground pipelines, heat exhausts and other infrastructure. Insufficient heat insulation of dormitories, on-campus apartments, classrooms and laboratories may cause an increased financial burden on UNI students. Therefore, such a study should be highly important for all campus residents.

### 2.2 Aerial Data

Aerial data was collected by the private contractor, AITScan (a division of Stockton Infrared Thermographic Services, located in Randleman, North Carolina) on April 04, 2007. The survey was performed between 11PM and 12AM to ensure maximum thermal contrast between the objects of interest (buildings, steam pipelines) and their surroundings [6, 7]. AITScan used the thermal infrared camera “Phoenix-Mid” manufactured by FLIR Systems. This camera has a resolution of 640 x 512 pixels with a FOV of 14.6°. With the average flying height of 600 m above the ground level, ground resolution is approximately 0.29 m for the whole dataset. This camera has a radiometric resolution of 14 bits and operates in the wavelength range of 3.0 - 5.0 μm. Cooling is provided by the on-board Stirling closed cycle cooler.

Imagery is provided by AITScan in two formats, JPEG and SAF (Standard Archive Format). The JPEG file format in not capable of storing original 14 bit data (maximum bit depth is 8 bit for the gray channel). SAF files, on the other hand, contain full 14 bit camera output (stored in 16 bit form for convenience), as well as camera calibration coefficients that are used to convert pixel values to temperature and radiance. The SAF file is, in essence, an archive, that stores a time series of imagery in the single-file form. This survey produced 24 SAF files, corresponding to the 24 flight lines. With each flight line consisting of approximately 250 separate images, the resulting dataset contains about 6,000 rasters.

### 2.3 Ground Data

**Figure 2.**
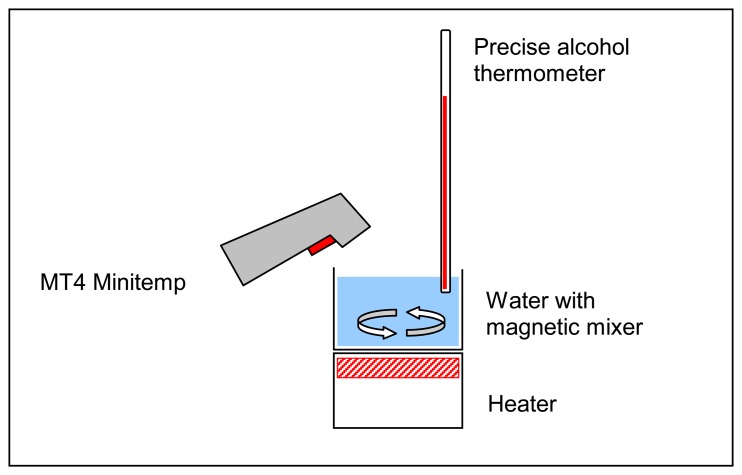
Infrared thermometer calibration.

In order to provide additional control over the results of the survey, ground-level temperature data was collected. A handheld infrared thermometer (“MT4 Minitemp” manufactured by RayTek) was used for ground-level temperature sampling. According to the manufacturer, the accuracy of “±2%, or ±1.7°C, whichever is greater,” can be achieved. Previous to taking any measurements, this thermometer was tested and calibrated in a laboratory setup, as illustrated by Figure 2.

A cup of cold water (around 1°C) was set up on a heater. A magnetic mixer at the bottom of the cup was constantly rotated by the magnetic field created by heater. The heater was turned on and as the temperature rose, simultaneous measurements were taken by the infrared and calibrated alcohol thermometer. Measurements were taken until the water reached 21°C. Then, the difference between two sets of temperatures was calculated. The differences were normally distributed with a mean of 0.4°C and standard deviation of 0.16. This test demonstrated that in the temperature range of 5°C to 20°C the accuracy was approximately ±0.3°C.

A number of random temperature measurements were taken around the campus, using GPS to record their positions. In addition to these samples, two reference targets - two sheets of plywood, 2.5 by 1.2 m, covered in black matte paint - were set up two hours before the start of the survey, approximately 1 m above the ground level, as far from expected sources of thermal pollution as possible [8]. These reference targets were later used in two different ways. First, both targets were used together with other ground samples to verify the pixel to radiance/temperature conversion model. In case the GPS positioning error would be too high to reliably identify the ground samples on the thermal imagery, two plywood targets would be a fall-back option to get at least some data for the model verification. Based on the proposed spatial resolution of 0.29 m, the size of the targets used would be enough for an accurate visual location. Second, the plywood targets were used to investigate the change of the temperature of the exposed objects overnight, if any. The sampling period was selected to match the data collection interval of the on-campus meteorology station.

Data from the on-campus automatic meteorology station located on the rooftop of the Latham Hall were collected every 30 minutes and contained temperature, pressure, and relative humidity values. Data on wind direction and speed were obtained from the nearby (6 km) meteorology station in Waterloo, Iowa. Throughout the night, the ambient air temperature stayed close to -6 °C, with humidity of 79%. Wind direction and speed were stable for the whole time of the survey (330°, 8 m/s). Except for the wind speed, meteorological conditions were favorable for the thermal infrared survey – clear sky, low ambient temperatures and absence of precipitation all benefit thermal contrast [6, 8].

### 2.4 GIS Data

Thermal imagery requires significant amount of processing. Automated means of analysis must be found in order to fully employ the benefits of remote sensing approach. Using GPS coordinates as a spatial reference, ground control samples were transformed into a shapefile. Next, the raster extraction tool from the ESRI ArcGIS toolbox was used to extract matching pairs of aerial and ground measurements. Another shapefile with on-campus buildings was prepared by digitizing a high- resolution raster image in ArcMap. This shapefile was used to analyze raster data separately for every building. Finally, a shapefile with the structure of the UNI steam pipeline network was provided by the facility services department. This shapefile was used to locate the segments of the steam network that were in any way obscured on the thermal image.

## 3. Methodology

### 3.1 Georeferencing and Mosaicing

As was mentioned before, aerial data was provided in the form of SAF files, which is an archive that stores a time series of images in the single-file form. In order to access individual raster images, another format had to be used. Portable Network Graphics format was chosen because it supports 16- bit grayscale data as well as lossless compression. An open-source software library, *libpng*, developed by Schalnat *et al.* [9] was used to create PNG rasters. Raw data was extracted from the SAF files with a small application written by the author of this paper in compliance with SAF specifications.

**Figure 3.**
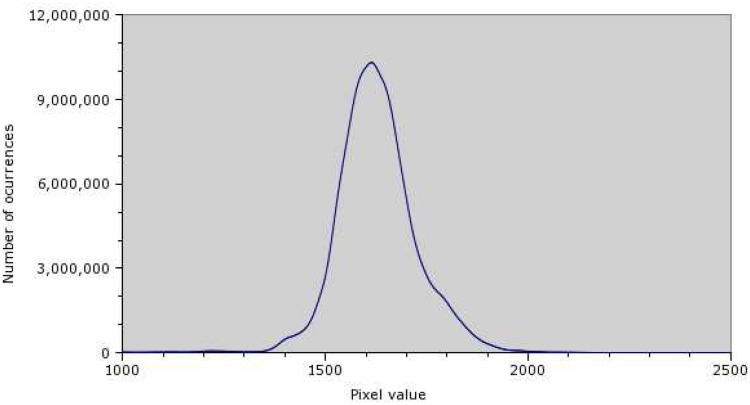
Pixel values distribution.

Before georeferencing and mosaicing, a histogram of the pixel values distribution was created (Figure 3).

**Figure 4.**
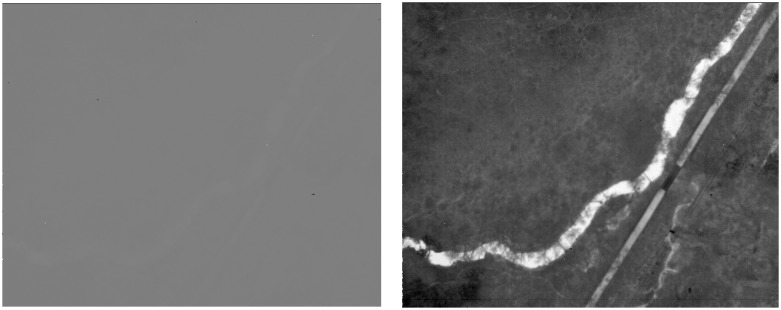
An image before (left) and after (right) the histogram stretch.

From the histogram, it is clear that the majority of pixels belong to the range of values between 1,300 and 2,100, which corresponds to less than 5% of the 16-bit range used by the camera. In order to improve visual contrast, the image histogram was stretched accordingly, using 850 and 2,835 as boundaries. Figure 4 is an example of an image before and after the histogram stretch. These boundary values were selected according to the Chebyshev's Inequality, which guarantees that 99% of the dataset are within the range specified.

After stretching the histogram, raster images were mosaiced manually in the ERDAS Imagine software using existing high-resolution airborne imageries of the UNI Campus as a reference. RMSE for the resulting model were normally distributed with mean of 1.94 pixels and standard deviation of 0.69.

### 3.2 Pixel to Radiance Conversion

There are two common ways to convert pixel values to temperature. The first approach, utilized by MacKay [2], Kulacki, Mintzer and Winget [3] and Tanis and Sampson [10] is to convert pixel values into temperature directly. The second approach is to convert pixel values to radiance, then use the radiance values as an input to the Planck's formula, as was done by Schott and Wilkinson [8] and Schott *et al.* [9]. In this study, the second approach was used, because it provides additional control over the level of emissivity of the objects of interest.

It was initially planned to convert pixel values into radiance by using conversion polynomials of the form

$$R=\sum_{i=0}^{n}c_{i}\cdot p^{i}$$

where c_i_ are calibration coefficients and p is the pixel value. Calibration data for the sensor was gathered on board the plane using a black body as a reference, as was done by MacKay [2], Tanis *et al.* [10] and Brown *et al.* [11]. However, the resulting polynomials produced erroneous temperatures that did not correspond to the ground truth. Instead, the dataset that was supposed to act as ground truth was used to create the regression model for the pixel to radiance conversion.

The regression model was built in two steps. First, using Planck's Law:

$$R(\lambda ,T)=\frac{B}{\lambda^{5}\cdot \left(e^{\frac{A}{\lambda T}}-1\right)}$$

where $A=\frac{hc}{k},B=2hc^{2}$, h is the Planck constant, k is the Boltzmann constant and c is the speed of light, the radiance of the ground samples was calculated as a function of their temperature. Only samples with well-known emissivity levels (concrete pavements) were used in this process. In the second step, ESRI ArcGIS was used to extract pixel values from the mosaiced thermal image at the locations of the ground samples. Then a linear regression model was built with the pixel values and radiance as variables.

$$R(p)=3.58838\cdot 10^{-4}\cdot p-0.290$$

The resulting model has a coefficient of determination (R^2^) of approximately 0.78. However, the model is only reliably defined for the range of pixel values between the coldest and the hottest object sampled (approximately -12°C and 2°C). Using the model produced in the steps above, as well as the inverted version of the Planck's Law,

$$T(\lambda ,R)=\frac{A}{\lambda \cdot \ln\left(\frac{B}{\lambda^{5}R}+1\right)}$$

pixel values were converted into temperatures. The level of emissivity was fixed at 0.95, which is the emissivity of concrete, according to the ASTER Spectral Library.

### 3.3 Model Validation

Some of the ground samples were left for the model validation process. Temperature estimates produced by the model were compared with the ground measurements.

## 4. Results and Discussion

### 4.1 Thermal Map of the UNI Campus

**Figure 5.**
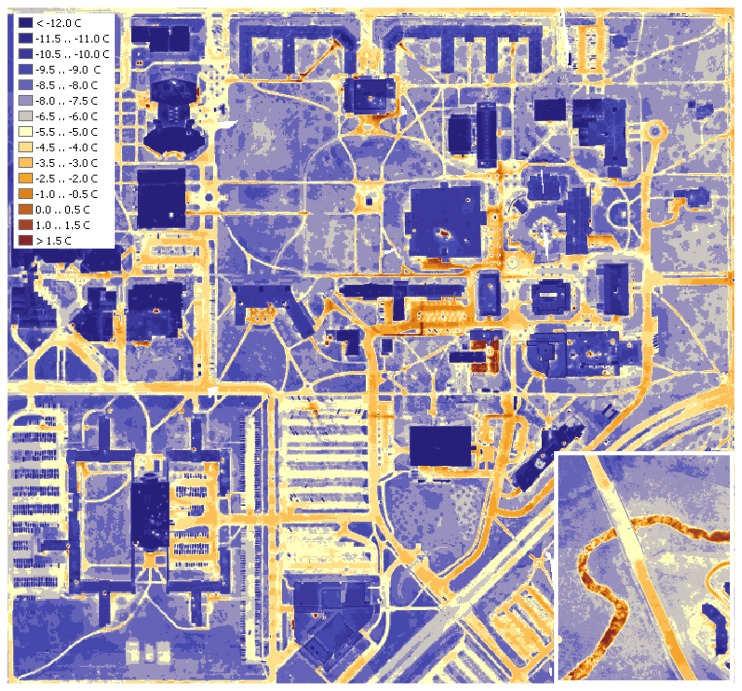
Thermal map of the campus.

Figure 5 shows the surface temperature variation map of the campus. This map clearly depicts the hot spots on the campus. Areas covered in vegetation tend to exhibit lower temperatures, while man- made pavements are relatively warm. Overall, places with lower height, tall vegetation or clusters of buildings tend to be warmer than high, open grounds, because they are better shielded from the cooling effect of the wind. Another easily spotted object is a small local stream (Figure 5, bottom right corner) - due to high thermal capacity of water, its warm stream is clearly outlined on the cool background. This study focused on two main objects of interest, on-campus buildings and underground steam pipelines mainly because of the facility services interests.

### 4.2 On-campus buildings

**Figure 6.**
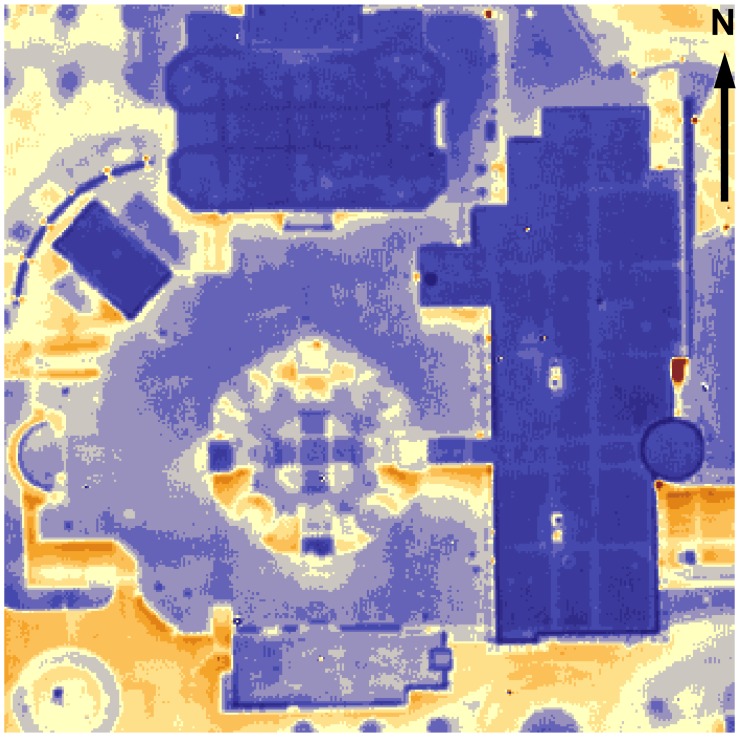
Maucker Union.

**Figure 7.**
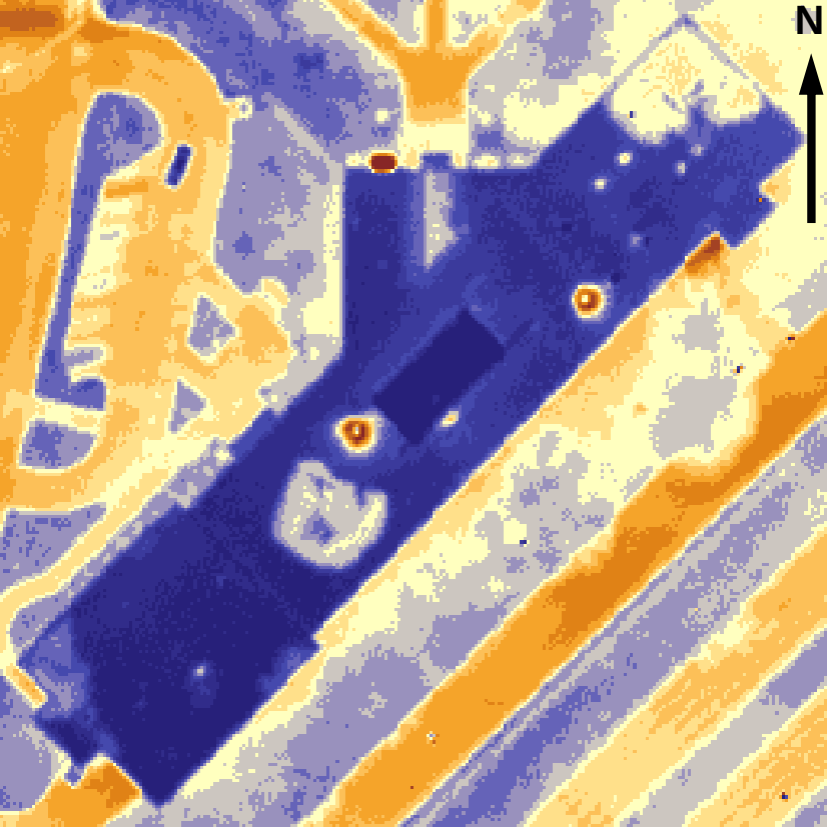
Latham Hall.

**Figure 8.**
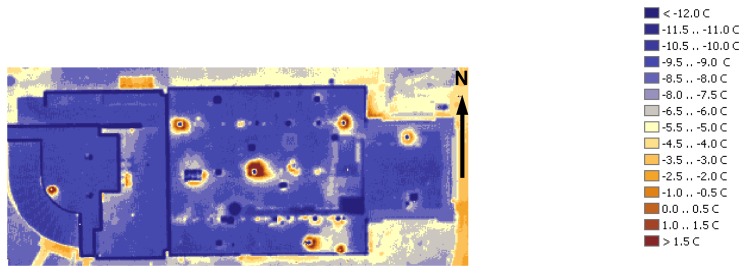
McColum Science Hall.

In general, rooftops of the on-campus buildings are in good condition with no major leaks visible. A number of buildings in this region have roof temperatures elevated above the average level, as well as other issues. For example, Maucker Union (Figure 6) has the warmest rooftop. Latham Hall (Figure 7) has a signature hotspot on its rooftop that can probably be attributed to the moisture, captured under the insulation material [4]. McColum Science Hall (Figure 8) hosts a number of chemistry and physics labs that are well-ventilated. The heat exhaust from the ventilation system is unmistakable, along with the overall warm rooftop of this hall.

For all buildings on campus, a so-called halo effect is present. Two surveys [6, 12] mention possible relationship between the walls temperature and the halo intensity. This effect is attributed to the reflection and re-emission of thermal radiation by the ground surface. Treado & Burch [12] attempted to derive the temperature of the object using its halo, but no clear relationship was found. Nonetheless, this effect serves the purpose of illustrating the wind-sheltering effect [8] of the on-campus buildings. The direction and the speed of the wind remained steady throughout the night (330°, 8 m/s). As a result, the areas to the south of the structures are noticeably warmer than their northern counterparts.

**Figure 9.**
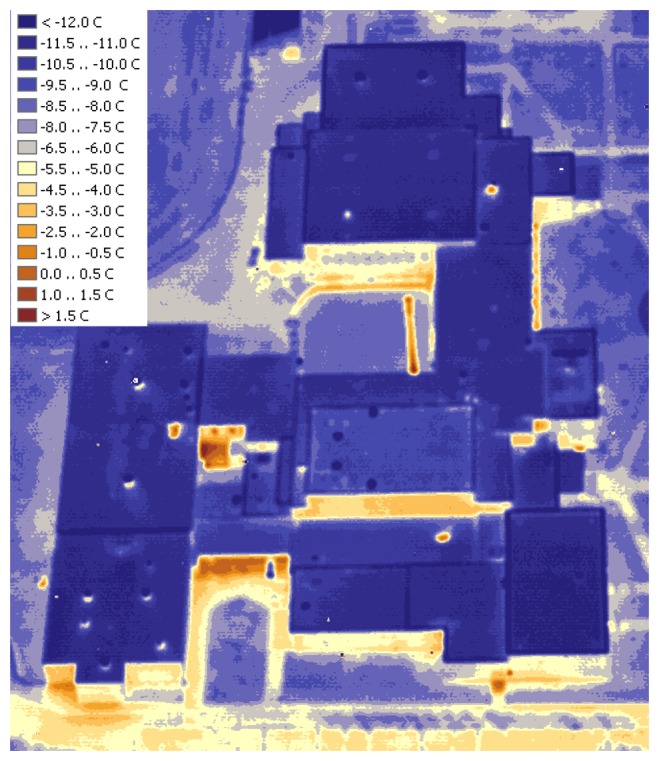
Oblique imagery.

As was mentioned before, the halo effect is poorly suited for analysis of wall temperatures. The study by Baraniak and Williams [1] is an example of a ground-based thermal imaging survey that deals with the building's walls directly. That study demonstrated that a number of significant temperature anomalies can be found by examining the structures from the side view. Because of the sharp turns the plane was performing at the end of each flight line, a number of oblique images of the buildings in the vicinity of the campus were produced. One of the best examples is present in the Figure 9. The building shown is Cedar Falls High School, located about 1 km to the north of the campus. The high visual contrast between the rooftop and the walls is obvious. No data on the insulation levels of this building was collected at the time of this paper being written, neither were there any ground samples available for that particular location. It should be noticed that factors other than temperature, such as the directional emissivity of the surfaces as well as wind effects contribute significantly to the observed contrast. Nonetheless, it would be useful to investigate the possibility of automated analysis of oblique thermal imagery in the future.

### 4.3 Steam pipelines

**Figure 10.**
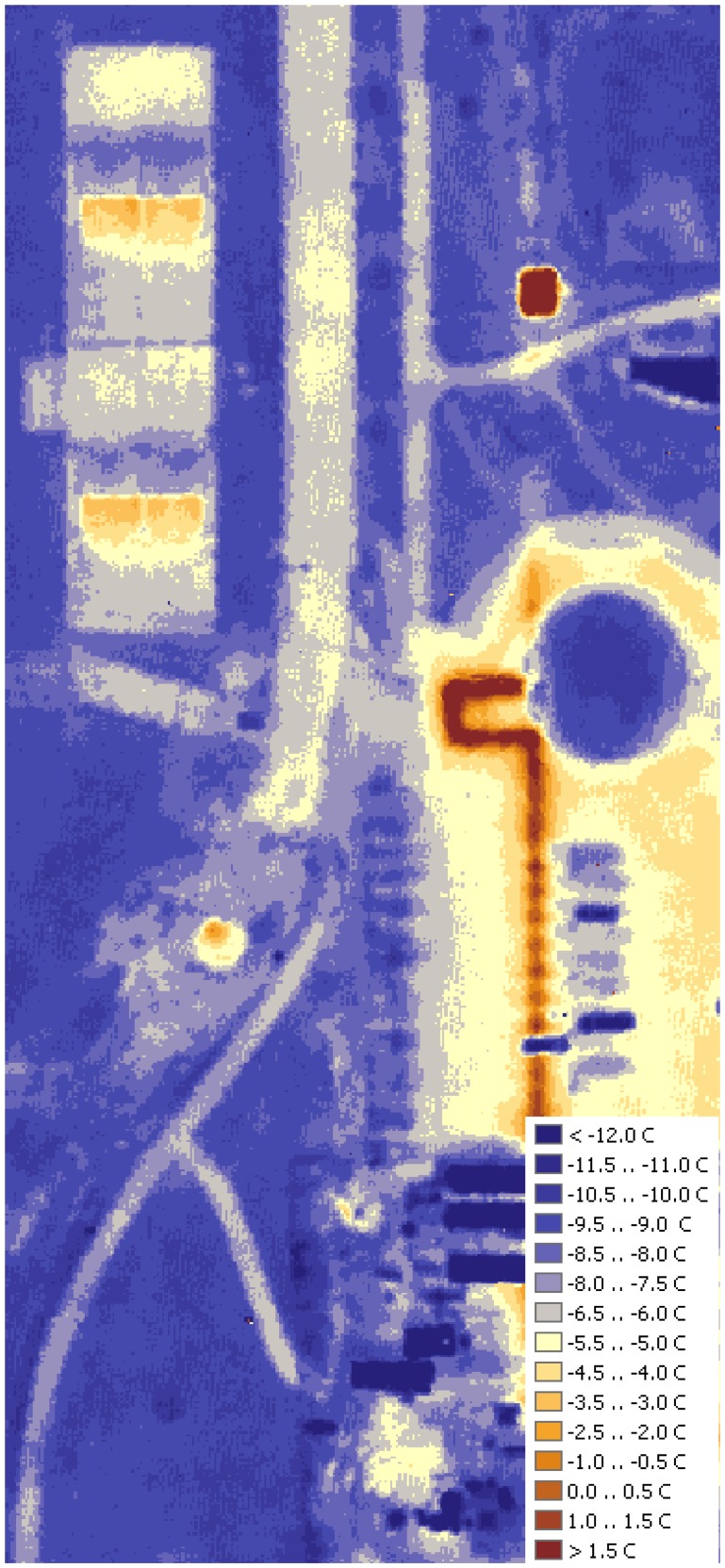
Pipelines and wind effect.

Several papers [2, 6, 8] illustrate that aerial infrared imagery is convenient and cost-efficient when heat loss from buried pipelines is analyzed. As shown by MacKay [2], pipeline locations must be known during the analysis because of the vast range of surface types, which may completely mask the thermal trace. Compared to other objects on campus, steam pipelines stand out most vividly (Figure 10). Combined with the shielding effect of the buildings, the heat coming from underground pipelines is the most possible explanation of the elevated temperatures in the eastern part of the campus. Estimating exact heat loss from underground pipelines requires precise knowledge of the burial depth and insulation levels of the pipeline. However, this information is usually obtained only after the line is dug out for maintenance or repair (most of the data available to the planning facilities of UNI is obsolete). However, thermal maps are still valuable as an excellent tool to locate segments of the steam network that require attention and, possibly, improvement.

### 4.4 Automated analysis

**Figure 11.**
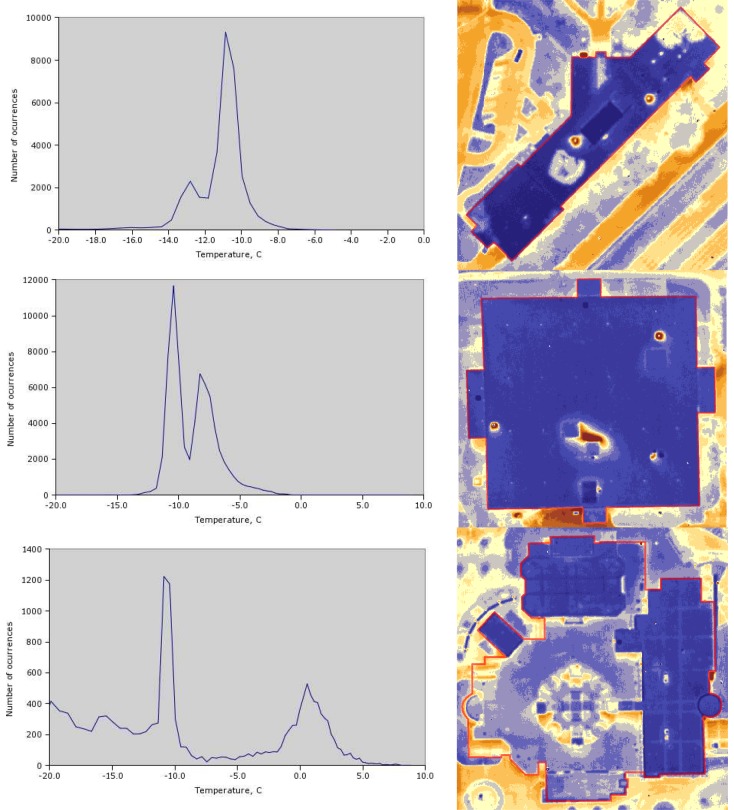
Individual Buildings' Temperature Histograms.

One of the issues not addressed in many papers is automated analysis of the imagery. As was mentioned in the Methodology section, a shapefile with vector representation of on-campus buildings was created. Using this shapefile and the extraction tool from the ESRI ArcGIS Toolbox, separate imagery datasets for each of the buildings were created. Pixel values from each dataset were extracted and used for simple statistical analysis, such as histogram plots. A number of examples are presented in Figure 11 (polygons from the shapefile are shown in red). Histogram peaks define the most frequent pixel values (or temperatures, for that matter). This approach allows for numerical comparison of the average temperatures between pairs of buildings automatically. For the UNI Campus the benefits are not so obvious, given the low number of buildings. For surveys of large residential areas, however, automation is important.

Temperature estimates produced by the model were compared with the ground measurements. The results of the validation are presented in Table 1. The mean temperature error is -0.25°C, standard deviation is 0.58°C. This makes the model accurate to within ±1.2°C.

## 5. Conclusions and Future Directions

In this study, a high-resolution thermal infrared map of the Campus of the University of Northern Iowa was produced. Inspection of the temperature map revealed a number of buildings that may be subject to insulation improvement due to their high rooftop temperature, as well as a number of smaller leaks, mostly related to ventilation exhaust. Most of the heat anomalies on the campus, however, are caused by poorly insulated steam pipelines. In particular, the large hot spot in the eastern of the campus is the result of high concentration of steam pipelines in the region as well as the shielding effect of the buildings. Overall, high-resolution thermal infrared imagery proved to be a highly useful tool for heat anomalies detection.

The observation conditions mentioned in the beginning of the paper – cold, dry and clear April night – played a critical part in the success of this survey. First of all, this ensured maximum thermal contrast between the objects of interest (buildings, steam pipelines) and their surroundings. Second, the combination of the low flying altitude and a cold dry atmosphere reduced the absorption and emission of the infrared energy by the column of air between the sensor and the objects of interest. In this paper, no corrections were made for the influence of the atmosphere, but this approach would not suffice for a survey with different observation conditions. A significant limitation of this study is absence of on- plane camera calibration. The ground control data was used to construct the pixel-to-temperature conversion model instead, which resulted in a much smaller validation dataset. Another limitation is that for the present moment, no correction for the emissivity values of the objects in question was done. An assumption was made that the UNI pavements fall into the generic “concrete” category mentioned in the ASTER Spectral Library, which would introduce a certain degree of error into the radiance to temperature conversion. Provided that the selection of the emissivity level does influence the derived temperature values directly, this is an important consideration. However, with the help of the Campus Planning Facilities, it would be possible to obtain the samples and exact types of all on- campus building materials, which would improve the accuracy of temperature retrieval. With the help of GIS data, emissivity correction can be applied to individual objects, instead of calibrating the whole map to a single level of emissivity. This work is reserved for a future paper. Finally, the oblique imagery obtained in this survey can be georeferenced and processed in the manner similar to the nadir imagery. As it was mentioned earlier, it would be necessary to account for the anisotropic nature of emissivity as well as the directional wind effects. This might be an opportunity to analyze the heat anomalies present in the buildings' walls.

## Figures and Tables

**Table 1.** Error Estimation.

**Model Temp.**	**Ground Temp.**	**Error**

-8.1	-8.1	0.00
-5.2	-5.0	-0.17
-8.1	-7.8	-0.28
-6.9	-6.7	-0.28
-8.1	-8.9	0.78
-5.8	-5.0	-0.78
-4.9	-3.9	-1.06
